# Effectiveness of social-therapeutic treatment for serious offenders in juvenile detention: A quasi-experimental study of recidivism

**DOI:** 10.3389/fpsyt.2022.909781

**Published:** 2022-10-20

**Authors:** Joscha Hausam, Melissa Franke, Robert J. B. Lehmann, Klaus-Peter Dahle

**Affiliations:** ^1^Institute of Forensic Psychiatry, Charité–Universitätsmedizin Berlin, Berlin, Germany; ^2^Department of Psychology, Medical School Berlin, Berlin, Germany; ^3^Department of Psychology, University of Hildesheim, Hildesheim, Germany

**Keywords:** social therapy, juvenile detention, therapeutic community, recidivism, offender treatment, effectiveness, propensity score matching, juvenile offenders

## Abstract

Due to legislative changes in Germany, there has been an increasing expansion of social-therapeutic facilities for juvenile offenders over the past 15 years. Social therapy comprises an eclectic mix of psychotherapeutic, educational, vocational, and recreational measures in a milieu-therapeutic setting to reduce recidivism of high-risk violent and sexual offenders. This study examined the effectiveness of social-therapeutic treatment on post-release recidivism among juvenile offenders. The sample included male offenders (*n* = 111) of the juvenile detention center in Berlin, Germany, aged 14–22 years, who were convicted of a violent (94%) or sexual offense (6%). Seventy-three subjects admitted to the social-therapeutic unit were compared to an offense-parallelized control group (*n* = 38) from the regular units using a propensity score based matching procedure. Initially, the groups did not differ with respect to risk (i.e., Level of Service Inventory - Revised) or risk-related characteristics (e.g., age). Subsequent Cox regression analyses revealed no average treatment effect on recidivism. Since the results indicated that the control group was not untreated, differential treatment effects were examined in a second step. School and vocational trainings had an effect on recidivism. The findings are discussed in light of the challenges in evaluating legally mandated offender treatment.

## Introduction

According to the latest police crime statistics, 16.7% of all registered crimes in Germany were committed by persons aged 14–21 years (1). While the majority of juveniles usually stop their delinquent behavior without criminal sanctioning, there is a small group showing persistent criminal behavior (2). According to the theory proposed by Moffitt the persistent delinquent behavior can be explained by a cumulative interaction of biological (e.g., neuropsychological health) and sociocultural (e.g., disadvantaged homes) factors culminating in a pathological personality (e.g., poor self-control, impulsivity). These persistent juvenile offenders are responsible for a majority of the serious crimes committed by their age group (3). The range of dispositional responses to juvenile delinquency includes educational measures, probation, and diversion. These informal measures are used in more than 70% of cases and are an effective means of preventing recidivism (4). Only when these options have been exhausted and proven ineffective detention is the last resort to prevent the perpetuation of a criminal career (5)^1^. However, it has been pointed out that there is little evidence that imprisonment reduces recidivism, but rather may have a criminogenic effect (6, 7). In Germany, two out of three detained juveniles recidivate after their release (8). Accordingly, effective juvenile offender treatment programs are needed to reduce recidivism (9).

Lipsey and Cullen (10) point out that correctional treatment generally has a better effect on juvenile offender recidivism than punishment (e.g., sanctions or supervision). Positive treatment effects were also reported for the subgroup of persistent juvenile offenders in correctional institutions (11). The most recent meta-review, including 48 meta-analyses and reviews from the past 40 years, similarly concludes that treatment programs can be an effective approach to reduce recidivism in juvenile offenders (12). These reviews emphasize that treatment effects vary depending on the criminal justice setting, program modalities, and offender type, among other factors. Roughly summarized, the largest effects are reported for institutionalized, structured, and well-implemented cognitive-behavioral treatment programs in serious (or high-risk) juvenile offenders (10–12). These findings align well with the risk, needs, and responsivity principles (RNR) of effective offender treatment, i.e., treatment should be tailored to each subject's risk level, criminogenic needs, thinking and learning patterns and should include cognitive-behavioral interventions (13). The RNR is empirically well established and has been shown to be effective for young offenders in previous research (14, 15). However, the majority of these studies come from the anglo-american area. In their meta-analysis of European studies with young offenders, Koehler et al. (14) report a significant mean effect in favor of treatment (Odds ratio = 1.34). Of the included studies, only 8% came from Germany. Thus, it can be stated that there is a lack of studies examining effectiveness of treatment with juvenile offenders.

### Social therapy in Germany

Social therapy represents the prototype of correctional treatment in Germany (16). In general, the primary correctional objective is resocialization, i.e., enabling offenders to live a socially responsible life in the future without committing further offenses. Social therapy is a specific, complex, and integrated correctional treatment approach to achieve this goal, particularly for violent and sexual offenders. One distinctive feature of social therapy is that it comprises an eclectic mix of psychotherapeutic, educational, vocational, and recreational measures in a milieu-therapeutic setting (17). The aim is to create a therapeutic climate in a residential group that provides a special social learning environment for the inmates. In this respect, it most closely resembles the concept of therapeutic communities in correctional facilities (18). In addition, the concept of integrative social therapy provides that the social environment in and outside the facility is considered and included in the treatment (17). It is important to note, however, that there is no such thing as “the” social therapy. Treatment methods and interventions can vary significantly between social-therapeutic facilities (16). As a result, an evaluation of the “total package” of social therapy is particularly challenging.

While the first social-therapeutic facilities for adult offenders opened as early as the 1970s, the last 15 years have seen an increasing number of social therapy units for juveniles, prompted by new legislation. Currently, there are 21 social-therapeutic facilities (out of 72 across Germany) for juvenile offenders, of which 15 facilities only started operating after 2006 (19). Admission to such a social-therapeutic unit is regulated by state law and is generally based on the “dangerousness” of an offender (e.g., Art. 20 of the Berlin Juvenile Court Act). Dangerousness is defined as the expectation of serious violent and sexual offenses in the future and is similar to the more common international concept of risk. Other juvenile detainees may also be placed in a social-therapeutic unit if treatment is indicated to achieve the correctional objective of resocialization.

Early evaluations of the effectiveness of social-therapeutic treatment in adult offenders indicated positive effects in the range of *r* = 0.10 (20). More recent, methodologically high-quality studies also show positive treatment effects (21, 22). In contrast, there are only a few studies on the effectiveness of social-therapeutic treatment in juvenile detention (23–26). Guéridon and Suhling (27) point out that these studies provide heterogeneous results and have some methodological weaknesses (e.g., lack of control group). In a preliminary study, social-therapeutic treatment had a positive effect on nonviolent recidivism but not on violent recidivism among police-supervised serious offenders (28).

On the one hand, the shortage of evidence is certainly due to the comparatively “young” social-therapeutic facilities in juvenile detention. On the other hand, evaluating routine correctional practice is a challenge for research. This is noteworthy because, as part of the above-mentioned amendments to the Juvenile Court Act, the effectiveness evaluation of treatment was enshrined in law (e.g., Section 103 of the Berlin Juvenile Court Act).

### Evaluation of correctional treatment

For several years, there has been intense debate about the best way to evaluate the effectiveness of offender treatment (29–31). The focus is primarily on the issue of internal validity, i.e., the extent to which the study design is free of bias and allows causal conclusions to be drawn about treatment effectiveness. The claim to conduct randomized controlled trials (RCTs) and thus to conduct research according to the “gold standard” (32, 33) encounters some barriers in the field of correctional treatment (e.g., random group assignment is difficult to reconcile with legally mandated treatments). In addition, RCTs in routine practice are also discussed critically (34–36). Quasi-experimental studies are rather common in evaluating offender treatment (37). These designs aim to estimate the effect of an intervention despite the lack of randomization, compromising internal validity. However, Ioannidis (38) shows that high-quality quasi-experimental studies can produce equivalent results to RCTs. This is supported by meta-analyses in which high-quality study designs do not influence treatment effect sizes (10, 39). According to Farrington et al. (40), studies are high quality if equivalence of control groups is ensured by statistical control or matching procedures. Propensity score matching (PSM) has recently become increasingly popular for estimating treatment effects in quasi-experimental or observational studies by matching treatment and control groups on a set of observed baseline covariates (41).

### Purpose of study

This study uses a quasi-experimental design and PSM to examine the effectiveness of social-therapeutic treatment in juvenile detention compared to an untreated control group. In a first step, the average treatment effect on post-release recidivism is examined. Social-therapeutic treatment is expected to have a significant positive effect on recidivism. In a second step, differential effectiveness of specific training and treatment measures is examined. This is done for two reasons. First, to disentangle the “total package” of social therapy. Second, some interventions are not offered “exclusively” in social therapy, but throughout the juvenile detention center. Since the law requires that juvenile detention has an “educational orientation” (e.g., § 3 of the Berlin Juvenile Court Act), it can be assumed that detainees outside of the social-therapeutic unit also receive treatment in some form.

## Methods

### Sample

The initial sample included 122 male juvenile and adolescent offenders admitted to the juvenile detention center Berlin between 2014 and 2016. Of these, 79 subjects were allocated to the social-therapeutic unit during November 2014 to July 2016. This was a full survey of all subjects enrolled during this period, there were no exclusion criteria. The remaining 43 subjects were an offense-parallelized sample admitted to the regular units during June to July 2016. Inclusion criteria were a juvenile sentence of at least 2 years for a violent or sexual offense and no indication for social therapy.

Eleven subjects had to be excluded from the analyses because recidivism data were not available^2^. Thus, the sample was *n* = 111, with 73 subjects in the treatment group (TG) and 38 subjects in the control group (CG). The average duration of detention in the TG was 36.98 months (*SD* = 13.44, Min–Max = 16.23–77.21) and 29.41 months in the CG (*SD* = 10.47, Min–Max = 11.70–67.45; *t*_(109)_ = −3.03, *p* < 0.001). In the TG, the average duration of treatment in the social-therapeutic unit was 23.09 months (*SD* = 10.65, Min–Max = 1.12–60.22). Twenty-one participants (28.8%) dropped out of treatment after an average of 17.04 months (*SD* = 17.04, Min–Max = 1.12–40.90)^3^. Of these, nine subjects (42.9%) were transferred back to the regular units of the juvenile detention center and 12 (57.1%) were transferred to an adult correctional facility for reasons of age. Following an intention-to-treat approach, dropouts remained in the TG for the analyses (43).

### Social-therapeutic unit

Opened in 2008, the social therapy is a separate unit within the juvenile detention center Berlin with a total of 50 treatment places and its own staff including detention officers, psychologists, and social workers. By law, primarily high-risk violent and sexual offenders are to be treated in the social-therapeutic unit. Allocation to this unit takes place in the diagnostic department. A formal admission criterion is a remaining juvenile sentence of at least 18 months and no more than 4 years. Contraindications include below-average intelligence, lack of language skills, predominant addiction problem, acute psychotic disorder, and lack of relationship skills, willingness to cooperate, or group skills.

Following the principles of integrative social therapy, the unit is organized as a therapeutic community and combines psychotherapeutic, educational, vocational, and recreational interventions^4^. According to its concept, treatment follows the RNR principles. Therefore, the primary goal is to address the criminogenic needs of high-risk offenders with structured, cognitive-behavioral interventions and attention to responsivity factors (e.g., motivation and learning style). After a 3-month intake and motivation phase, the treatment phase includes weekly individual psychological sessions and three manualized groups: The Reasoning and Rehabilitation program consisting of 35 dual hours [R&R (44)], an adapted version of the Violent Offender Therapeutic Program with 53 sessions (45), and an adapted version of the Sex Offender Treatment Program with 77 sessions (46). The implementation and regularity of these individual and group interventions is a key difference from the regular units, enabled with better staffing in the social-therapeutic unit (e.g., twice the number of psychologists per detainee). The treatment lasts at least 12 months. In the final discharge phase, the goal is to gradually reintegrate the participants back into the community. Treatment can be discontinued if there is a lack of motivation, willingness to participate, the rules of the unit are repeatedly and significantly violated, or the treatment goals of other participants are endangered. In this case, relocation to a regular unit of the juvenile detention is indicated.

### Data collection and measures

This study is part of an evaluation project commissioned by the Berlin Senate for Justice, Consumer Protection and Anti-Discrimination. Data were collected based on the inmate file at the respective time of release and the Berlin police database in November 2021. Ethical approval for the study was sought and granted by the Ethics Committee of Charité - Universitätsmedizin Berlin (EA4/131/18). All participants gave written informed consent in accordance with the Declaration of Helsinki and the EU General Data Protection Regulation. The protocol was approved by the Official Data Protection Officer of Charité. Parental consent was obtained for five subjects who were younger than 16 years at the time of data collection.

#### Pretreatment characteristics

The following characteristics were coded based on detainee files: German nationality (yes/no), migrant background (yes/no), school graduation (yes/no), previous conviction for a violent or sexual offense (yes/no), age at first conviction, age at detention, length of current juvenile sentence, and type of current offense (homicide, robbery, assault, or sexual offense).

In addition, professionally trained research assistants applied the Level of Service Inventory-Revised [LSI-R (47); German version (48)] as a risk measure based on complete youth records of the subjects. The predictive validity of the LSI-R is well documented, also with young offenders (49) and in German speaking samples (50).

#### Training and treatment

We recorded which training and treatments detainees in both groups participated in. Regarding training measures, we coded whether a person completed educational or vocational training (or these interventions were ongoing at the time of discharge). Further, we recorded whether participants attended at least one of the following treatment interventions during detention: Individual psychological counseling, R&R program, violence prevention, social skills training, and addiction group. Of the sample, none participated in the sex offender group program, so that is not included here. Please recall that some interventions are offered exclusively (e.g., R&R) or much more intensively in the social-therapeutic unit (e.g., multi-week structured group for violent offenders in the social-therapeutic unit vs. multi-day anti-aggression training in regular units).

#### Recidivism

Post-release recidivism was obtained from police records. These records capture whether the police accused or apprehended a person being a primary suspect of an offense. Importantly, these constitute neither charges nor convictions and only include offenses committed in the state Berlin. The follow-up period was significantly longer in the TG (*M* = 60.85 months, *SD* = 16.23, Min–Max = 24.25–85.19) than in the CG (*M* = 52.30 months, *SD* = 10.58, Min–Max = 20.63–65.18; *t*_(103.44)_ = −3.34, *p* < 0.01, *d* = 0.59). We coded the absence/presence and time of a non-violent/non-sexual (e.g., thievery, drug offense), violent (e.g., robbery, assault), and sexual offenses (e.g., sexual abuse, rape). Because of low base rates of sexual recidivism in both the TG (*n* = 4) and CG (*n* = 2), violent and sexual recidivism was collapsed into one category. Rates in the sample were 81.1% (*n* = 90) for non-violent/-sexual recidivism and 51.4% (*n* = 57) for violent/sexual recidivism.

### Data analysis

Data analysis was performed using R version 4.12 (51) and the packages “MatchIt” [v3.3.3 (52)] and “survival” [v3.2-13 (53)]. The matching procedure is described first, followed by the subsequent statistical analyses and the results of a power analysis.

#### Propensity score matching

Based solely on the legal requirement that primarily high-risk violent and sexual offenders are to be treated in the social therapeutic unit, a priori differences between the TG and CG must be assumed. Since it was not possible to conduct a randomized controlled trial in the present study - as is often the case in evaluations of correctional treatment - statistical methods were used to account for this selection bias. PSM was applied to compare subjects who were treated in the social-therapeutic unit with those who were not. PSM is a commonly used analytic method for estimating treatment effects that “allow one to mimic some of the characteristics of an RCT in the context of an observational study” [(41), p. 400]. Simulation studies indicate that PSM can be used in small samples to obtain correct estimates of treatment effect (54).

Technically, the propensity score is defined as the probability of being assigned to a treatment group based on a certain set of (pretreatment) characteristics. PSM consists of several steps: Covariate selection, propensity score estimation, matching specification, and assessment of covariate balance (41). Covariate selection is a critical component of PSM and the optimal approach is the subject of ongoing scientific debate (55, 56). We chose to include variables that are theoretically associated with both treatment and recidivism rather than based on preliminary statistical testing (41). A total number of 12 variables (see Table 1; index offense variable was dummy coded) was selected as covariates. The propensity score was estimated using logistic regression analysis, with treatment as criterion and the covariates as predictors. Based on the propensity score, we used a full matching procedure, primarily because it does not discard any cases and it provides an estimate of the average treatment effect (57, 58). In full matching, subgroups are formed consisting of either one treated and at least one untreated subject or one untreated and at least one treated subject. While full matching is referred to as a matching procedure, it is actually a combination of matching, stratification, and weighting: Strata are formed consisting of treated and control subjects, and the weights resulting from the stratification are then included in subsequent analyses (57, 59).

**Table 1:** Pretreatment characteristics of the control (*n* = 38) and treatment group (*n* = 73).

	**Control**	**Treatment**	**χ^2^**	***p***	***V***
	**% (*n*)**	**% (*n*)**			
German nationality	71.1 (27)	60.3 (44)	1.26	0.262	0.11
Migrant background	76.3 (29)	82.2 (60)	0.54	0.461	0.07
School diploma	34.2 (13)	35.6 (26)	0.02	0.883	0.01
Criminal record with violent or sexual offense	78.9 (30)	83.6 (61)	0.36	0.548	0.06
**Index offense**					
- Homicide	10.5 (4)	11.0 (8)	1.08	0.781	0.10
- Robbery	60.5 (23)	50.7 (37)			
- Assault	23.7 (9)	31.5 (23)			
- Sexual offense	5.3 (2)	6.8 (5)			
	***M*** **(*SD*)**	***M*** **(*SD*)**	***t***	***p***	***d***
Index sentence length (months)	38.18 (14.72)	40.32 (12.50)	−0.80	0.425	0.16
Age of onset	15.87 (1.74)	15.33 (1.47)	1.72	0.088	0.34
Age at index	18.37 (2.10)	18.65 (1.74)	−0.75	0.453	0.15
LSI-R	24.13 (8.20)	26.04 (5.80)	−1.42	0.158	0.29

V, Cramer's V effect size; d, Cohen's d effect size; LSI-R, level of service inventory– revised.

Finally, covariate balance was assessed before and after matching. It indicates the extent to which the distribution of covariates is similar across groups. As shown by univariate balance summary statistics and visual diagnostics, covariate balance improved (see Supplementary material 1). After matching, all standardized mean differences for the covariates were close to or below 0.1, variance ratios were closer to 1, and empirical cumulative density functions (eCDF) were closer to 0. Visual diagnostics such as eCDF plots, empirical quantile-quantile (eQQ) plots, and kernel density plots also indicate improved covariate balance after matching. Following the recommendations of Ho et al. (60), it was concluded that balance between TG and CG is adequate (but not perfect).

#### Statistical analysis

Group comparisons were calculated to examine differences in pretreatment characteristics and intervention and treatment participation (*t*-tests or chi^2^ tests depending on the type of variable). In addition, Cohen's *d* or Cramer's **V** effect sizes are reported.

Subsequent survival analyses are based on the matched groups. Austin and Stuart (57) describe the use of full matching with survival outcomes. Cox proportional hazards models were estimated to examine the time-dependent recidivism course of individuals in both groups, taking into account the different follow-up times. Two models were calculated for each of the two recidivism outcomes. Robust uncertainty estimation (i.e., of standard errors, *p*-values, and confidence intervals) was used.

First, to assess the average treatment effect, univariate Cox models were estimated by regressing the outcome on the treatment weighted by the matching weights and including the formed subgroups as clusters. In a similar way, secondly, multivariate cox regression models were calculated to examine the effects of the interventions and treatment measures. Importantly, in the first two models, the hazard ratio (HR) corresponds to the marginal effect of treatment, and in the other two, HR reflects conditional effects. Conditional effects denote an average effect at the individual level, while marginal effects denote a population-level effect (61).

There were no outliers in the sample and the assumption of proportional hazards was met in all models (according to Schoenfeld residuals, see Supplementary material 2).

#### Power analysis

A statistical power analysis was performed to estimate sample size, based on the meta-analysis by Koehler et al. (14) and the effect size of OR = 1.34. With an alpha = 0.05 and power = 0.80, the projected sample size needed with this effect size is approximately *N* = 267 for a Cox proportional hazards model. Since duration of data collection was limited in time by the client as indicated above, the sample may be too small to find the expected effect. Therefore, we performed a *post-hoc* power analysis to examine the power of subsequent analyses. Using the same alpha, available sample size (*n* = 111), expected effect size, and an adjustment for censoring (base rates for both recidivism criteria), a power of 0.49 for non-violent/sexual recidivism and of 0.35 for violent/sexual recidivism was determined. Thus, the probability of a type II error is increased in the present study.

## Results

There were no significant group differences in pretreatment characteristics between CG and TG (see Table 1). Indicated by small (but non-significant) effects, however, subjects in the TG were younger at onset (*d* = 0.34) and had a slightly higher LSI-R total score (*d* = 0.29).

A more differentiated picture emerged for intervention and treatment participation (see Table 2). In both groups, about one in four to one in five subjects completed either school graduation or vocational training (or the intervention was still ongoing at the time of discharge), with no significant differences. Similarly, there were no differences between TG and CG in participation in the social skills training (23.3 vs. 34.2%), violence prevention (28.8 vs. 23.7%), and addiction treatment groups (34.2 vs. 36.8%). However, the subjects in the TG attended more frequently individual psychological sessions [98.6 vs. 44.7%; χ^2^(1) = 45.68, *p* < 0.001, *V* = 0.64] and the R&R group [67.1 vs. 0%; χ^2^(1) = 45.67, *p* < 0.001, *V* = 0.64].

**Table 2:** Intervention and treatment participation in the control (*n* = 38) and treatment group (*n* = 73).

	**Control**	**Treatment**	**χ^2^**	***p***	***V***
	**% (*n*)**	**% (*n*)**			
Educational training	15.8 (6)	20.5 (15)	0.37	0.544	0.06
Vocational training	23.7 (9)	26.0 (19)	0.07	0.787	0.03
Individual psychological treatment	44.7 (17)	98.6 (72)	45.68	< 0.001	0.64
R&R program	0 (0)	67.1 (49)	45.67	< 0.001	0.64
Social skills training	34.2 (13)	23.3 (17)	1.51	0.219	0.12
Violence prevention group	23.7 (9)	28.8 (21)	0.33	0.567	0.05
Addiction group	36.8 (14)	34.2 (25)	0.07	0.786	0.03

V, Cramer's V effect size; R&R program, reasoning and rehabilitation program.

Rates of recidivism are shown in Table 3. There were no significant differences between TG and CG in non-violent/-sexual recidivism (82.2 vs. 78.9%) and violent/sexual recidivism (53.4 vs. 47.4%). Noteworthy, there were no statistically significant differences in recidivism with respect to the index offense and between completers and dropouts in the TG. Dropouts showed a weak tendency to relapse more frequently (90.5 vs. 78.8% for non-violent/-sexual and 57.1 vs. 51.9% for violent/sexual recidivism).

**Table 3:** Post-release recidivism in the control (*n* = 38) and treatment group (*n* = 73).

	**Control**	**Treatment**	**χ^2^**	***p***	***V***
	**% (*n*)**	**% (*n*)**			
Non-violent/-sexual recidivism	78.9 (30)	82.2 (60)	0.17	0.679	0.04
Violent/sexual recidivism	47.4 (18)	53.4 (39)	0.37	0.545	0.06

V, Cramer's V effect size.

In the univariate Cox models, no average treatment effect was evident for either non-violent/-sexual recidivism [LR-χ^2^(1) = 1.35, *p* = 0.2] or violent/sexual recidivism [LR-χ^2^(1) = 0, *p* = 1]. The hazard ratio for non-violent/-sexual recidivism was slightly lower in the TG (HR = 0.76) and for violent/sexual recidivism it was almost identical to that of the CG (HR = 1.01), although neither was significant (see Table 4).

**Table 4:** Average treatment effects assessed with full matching procedure in univariate cox regression analyses (*n* = 111).

	**HR**	***SE***	***z***	***p***	**95% CI**
Non-violent/-sexual recidivism	0.76	0.24	−1.15	0.249	0.47, 1.22
Violent/sexual recidivism	1.01	0.31	0.03	0.979	0.55, 1.84

HR, hazard ratio; SE, standard error; CI, confidence interval.

The results of the multivariate Cox models are shown in Table 5. Neither the model for non-violent/-sexual recidivism [LR-χ^2^(7) = 7.14, *p* = 0.4] nor violent/sexual recidivism was significant [χ^2^(7) = 11.74, *p* = 0.1]. A significant conditional effect of vocational training on severe recidivism (HR = 0.69, *p* < 0.01) and a marginally significant effect of school training on non-violent/-sexual recidivism (HR = 0.87, *p* = 0.063) was found.

**Table 5:** Conditional effects of intervention and treatment participation assessed with full matching procedure in multivariate cox regression analyses (*n* = 111).

	**HR**	***SE***	***z***	***p***	**95% CI**
**Non-violent/-sexual recidivism**					
Educational training	0.87	0.08	−1.86	0.063	0.75, 1.01
Vocational training	0.89	0.08	−1.40	0.162	0.75, 1.05
Individual psychological treatment	0.71	0.29	−1.16	0.246	0.40, 1.26
R&R program	0.85	0.28	−0.58	0.564	0.49, 1.48
Social skills training	1.03	0.23	0.12	0.905	0.66, 1.60
Violence prevention group	0.98	0.23	−0.10	0.921	0.62, 1.54
Addiction group	1.01	0.20	0.08	0.940	0.69, 1.51
**Violent/sexual recidivism**					
Educational training	0.88	0.13	−1.00	0.316	0.71, 1.23
Vocational training	0.69	0.14	−2.62	0.001	0.53, 0.95
Individual psychological treatment	1.08	0.41	0.18	0.857	0.47, 2.46
R&R program	0.99	0.31	−0.05	0.962	0.52, 1.32
Social skills training	1.31	0.37	0.73	0.463	0.71, 2.67
Violence prevention group	1.00	0.36	−0.01	0.991	0.48, 1.83
Addiction group	1.07	0.31	0.22	0.826	0.67, 1.79

HR, hazard ratio; SE, standard error; CI, confidence interval; R&R program, reasoning and rehabilitation program.

## Discussion

Due to legal changes in Germany, there has been a strong growth in social-therapeutic facilities for the treatment of serious juvenile offenders in recent years. These institutions are based on the concept of integrative social therapy, for which positive results have been reported in the adult correctional system (16). Studies on the effectiveness of social therapy in juvenile detention are rare. Therefore, the aim of the present study was to compare post-release recidivism in a group of young violent and sexual offenders who received social-therapeutic treatment with an offense-parallelized control group of juvenile detainees. The groups were compared using a propensity score based full matching procedure (57, 59, 62).

No average treatment effect was found for both recidivism criteria. Recidivism rates were comparably high in both groups. This result is contrary to our expectations, considering that the investigated social-therapeutic unit includes essential features that have been shown to be effective in juvenile offender treatment [e.g., cognitive-behavioral interventions with high-risk offenders (10–12)]. Further, it is not consistent with the results of a preliminary study, which found a treatment effect on non-violent recidivism (28). Therefore, some aspects that may have affected the results should be considered here.

First, by law, high-risk juvenile offenders are primarily to be treated in the social-therapeutic unit. This is in line with the risk principle of effective offender treatment, according to which treatment intensity should be based on risk (13). However, the analyses showed that there were hardly any group differences, neither in risk (i.e., LSI-R score) nor in risk-relevant characteristics (e.g., age). One explanation could be that the diagnostic unit did not strictly adhere to the risk principle. Intensive treatment, such as social therapy, that is not following the risk level is less effective or may even have adverse effects in the case of low-risk offenders (13). However, this is contradicted by the result that more than half of both groups in the sample posed at least a moderate-high risk. Another explanation could be that the treatment assignment was “correct” but we were unable to reflect these risk differences in our data. In this context, Dahle and Schmidt (50) reported that the LSI-R had unsatisfactory predictive validity among young offenders with a migrant background from a predominantly Muslim culture. Similar results have been reported for other ethnic groups (49, 63). Approximately 80% of our sample had a migrant background. Hence, it would be possible that the LSI-R was not an appropriate choice in the present study (64). Possible unidentified group differences would not have been controlled for in the matching procedure and thus would have systematically affected the results.

Second, not all participants received the social-therapeutic treatment as planned. The dropout rate in our study is largely consistent with international (65) and national findings (66, 67). Olver et al. (65) also note that dropouts are at higher risk before treatment and have higher recidivism rates after release than completers. Thus, treatment dropout may be a confounding characteristic in many cases. Another hypothesis regarding this mechanism is that treatment attrition may further increase the risk of recidivism compared to no treatment (68). We included dropouts in the analyses to reduce this methodological bias and to obtain a more accurate estimate of treatment effectiveness as delivered in routine practice (43). Nevertheless, it may be assumed that the inclusion of dropouts had an impact on the results. Our preliminary analyses also suggest a dropout effect; at the very least, recidivism was slightly higher. More differentiated analyses of dropouts and completers should be conducted in the future to provide relevant information on treatment efficacy, appropriateness of treatment assignments, and obstacles inherent to treatment (67).

Third, the results showed that the control group was not untreated. This corresponds to the legal requirement for an “educational orientation” of juvenile detention. The subjects of both groups equally participated in school and vocational trainings as well as some treatment groups (e.g., social skills group). There were differences only in participation in individual psychological sessions and in the R&R group (the latter actually being offered only in the social-therapeutic unit). Thus, trainings and treatment may also had a recidivism-reducing effect in the control group. We explored this question with further analyses.

A second objective of this study was to examine differential effects of training and treatment measures throughout the juvenile detention center. The multivariate Cox regression analyses showed a significant effect for vocational training. Accordingly, participants who had completed vocational training in detention or who were still in vocational training at the time of release had a 31% reduced risk of violent/sexual recidivism. In addition, a marginally significant effect was found for school training on non-violent/-sexual recidivism. This is consistent with meta-analytic evidence and reinforces the importance of school and vocational training in juvenile detention (14, 69).

### Limitations

Comparison of our results with previous studies on juvenile offender treatment may be limited by differences such as legislature, treatment context, and sample characteristics. These may have affected external validity. For example, four in five of the juvenile offenders in our sample had a migration background whereas only one in four has such a background in the general population in Germany. Even though this point could not have affected our results as both samples were matched future studies might want to investigate the social features of juvenile offenders in a therapeutic context in more detail. Furthermore, some methodological limitations have to be taken into account. First, the small sample (especially the control group) and the reduced power of the analyses must be considered. More precisely, it is to be expected that an actual effect was statistically not found. Second, the quasi-experimental design is particularly noteworthy. Propensity score matching can produce valid results even in small samples (54), but the analyses are always based on observed variables only. Therefore, other characteristics not included in the present study could have a significant influence. Because future evaluations will continue to be mostly quasi-experimental, there is a need for studies that examine what cofounded variables should be considered in matching procedures in the context of offender treatment. Third, diverse offense groups were examined together. These did not differ in recidivism, but it is still conceivable that offender types react differently to social-therapeutic treatment. Heterogeneous treatment effects should be examined in more detail. Another limitation relates to the coding of treatment participation. While the coding of educational and vocational trainings implied a certain level of intensity and success (completed or ongoing), for all other treatments, a single participation was already assessed. Since the intensity or success of treatment participation is central to the recidivism-reducing effect, the operationalization may have led to insufficient differentiation. This could be an explanation for the lack of conditional effects. In addition, we did not assess treatment integrity (70). Finally, future studies should consider additional recidivism data with similarly long follow-up periods to make more robust conclusions about the effectiveness of social-therapeutic treatment. The outcomes used here are based on police proceedings, of which we do not know whether and how they were further proceeded (e.g., to court). Beyond that, they do not allow any statement about recidivism severity. Another possible bias is that only offenses in the state of Berlin, and not all of Germany, were considered. As part of our ongoing project, we will soon be able to collect recidivism data based on criminal records and conduct more sophisticated analyses.

## Conclusion

With the increase in social-therapeutic units in juvenile detention and the demand for evidence-based practice, evaluations of treatment effects in routine practice are very important, but also challenging. The present quasi-experimental study did not find sufficient evidence for an average treatment effect of social-therapeutic treatment in juvenile detention. As outlined, however, our results should be interpreted with caution. Among other things, the results showed that the control group was not untreated. Subsequent analyses revealed significant effects of educational and vocational training on recidivism in the overall sample. On the one hand, this underscores that it may be worth examining for differential effects of specific training and treatment interventions (rather than looking exclusively at average treatment effects). This seems to be especially important for correctional treatment as complex and comprehensive as social therapy. On the other hand, the results raise the question of whether the higher costs of social-therapeutic treatment are justified. Future research should therefore address the extent to which specific social-therapeutic treatment, beyond the interventions usually provided in juvenile detention (e.g., educational or vocational training), affect recidivism. There will continue to be obstacles in presenting causal treatment effects of the “total package” of social therapy. Nevertheless, more replications are needed (71).

## Data availability statement

The datasets presented in this article are not readily available because this study is part of an evaluation project commissioned by the Berlin Senate for Justice, Consumer Protection and Anti-Discrimination. We do not have the right to disclose the data. Requests to access the datasets should be directed to poststelle@senjustva.berlin.de.

## Ethics statement

The studies involving human participants were reviewed and approved by Ethics Committee of Charité - Universitätsmedizin Berlin, Germany (EA4/131/18). Written informed consent to participate in this study was provided by the participants or participants' legal gurdian/next of kin.

## Author contributions

JH and MF designed the study. MF collected data. JH performed statistical analyses and wrote the first draft of the manuscript with support of MF and RL. K-PD supervised the project. All authors contributed to manuscript revision, read, and approved the submitted version.

## Funding

The authors disclosed receipt of the following financial support for the research, authorship, and/or publication of this article: The evaluation project was funded by the Senate for Justice, Consumer Protection and Anti-Discrimination of Berlin, Germany.

## Conflict of interest

The authors declare that the research was conducted in the absence of any commercial or financial relationships that could be construed as a potential conflict of interest.

## Publisher's note

All claims expressed in this article are solely those of the authors and do not necessarily represent those of their affiliated organizations, or those of the publisher, the editors and the reviewers. Any product that may be evaluated in this article, or claim that may be made by its manufacturer, is not guaranteed or endorsed by the publisher.

## Footnotes

1 This age span is covered by the German Juvenile Justice Act. Children under the age of 14 are not held as criminally responsible. Juveniles (14–17 years) must, and young adults (18–21 years) can be judged by juvenile law. Unlike other countries (e.g., United States), more than 80% of young adults are sentenced by juvenile law for the most serious crimes (4). For simplicity, we will refer to both age groups as “juveniles” in this study.

2 Nine subjects were deported to their country of origin due to lack of residence permit and two cases could not be clearly identified in the police database. With regard to the pretreatment characteristics (Table 1), there were significant differences between excluded and included subjects only for German nationality (9.1 vs. 64.0%; *p* < 0.001) and sentence length (51.55 vs. 39.59 months; *p* < 0.01).

3 A preliminary study found that treatment dropouts are initiated in almost all cases by the social-therapeutic unit mostly due to rule violations (60.5%), lack of treatment motivation (51.2%), lack of therapeutic relationship (25.6%), and/or jeopardizing the correctional goal of other inmates [39.5%; (42)].

4 It should be noted that the social-therapeutic unit utilizes institution-wide resources. This primarily concerns school, vocational, and recreational measures as well as counseling (e.g., debt counseling) and aftercare services.
