# Supplementary material for: Effectiveness of social-therapeutic treatment for serious offenders in juvenile detention: A quasi-experimental study of recidivism
Source: Front Psychiatry. 2022 Oct 20;13:909781. doi: 10.3389/fpsyt.2022.909781 (PMC9631022; doi:10.3389/fpsyt.2022.909781)
Supplement: Supplementary file 1 [file Data_Sheet_1.PDF]

frontiers

Electronic Supplementary Material (ESM)

ESM 1: Covariate balance before and after full matching

Summary of Balance for All Data:

	Means Treated	Means Control	Std. Mean Diff.	Var. Ratio	eCDF Mean	eCDF Max
distance	0.6906	0.5943	0.6659	0.6125	0.1813	0.3450
german_nationality	0.6027	0.7105	-0.2285	.	0.1078	0.1078
migrant_background	0.8219	0.7632	0.1453	.	0.0588	0.0588
school	0.3562	0.3421	0.0295	.	0.0141	0.0141
criminal_record	0.8356	0.7895	0.1184	.	0.0461	0.0461
index_homicide	0.1096	0.1053	0.0140	.	0.0043	0.0043
index_robbery	0.5068	0.6053	-0.1991	.	0.0984	0.0984
index_assault	0.3151	0.2368	0.1757	.	0.0782	0.0782
index_sex	0.0685	0.0526	0.0665	.	0.0159	0.0159
age_index	18.6497	18.3684	0.1461	0.6853	0.0600	0.1763
age_onset	15.3288	15.8684	-0.3345	0.7141	0.0540	0.1446
index_sentence	40.3151	38.1842	0.1560	0.7216	0.1030	0.2300
lsi_score	26.0411	24.1316	0.2690	0.4995	0.0701	0.1651

Summary of Balance for Matched Data:

	Means Treated	Means Control	Std. Mean Diff.	Var. Ratio	eCDF Mean	eCDF Max	Std. Pair Dist.
distance	0.6592	0.6558	0.0230	0.9524	0.0192	0.0721	0.0802
german_nationality	0.6423	0.6622	-0.0421	.	0.0198	0.0198	1.0196
migrant_background	0.7899	0.7913	-0.0033	.	0.0014	0.0014	0.8451
school	0.3588	0.3273	0.0661	.	0.0315	0.0315	1.1154
criminal_record	0.8225	0.8483	-0.0663	.	0.0258	0.0258	0.7798
index_homicide	0.1069	0.1021	0.0155	.	0.0048	0.0048	0.6132
index_robbery	0.5594	0.5751	-0.0316	.	0.0156	0.0156	0.8449
index_assault	0.2740	0.2748	-0.0016	.	0.0007	0.0007	0.9381
index_sex	0.0596	0.0480	0.0485	.	0.0116	0.0116	0.4779
age_index	18.5646	18.5045	0.0312	0.8591	0.0472	0.1635	0.9666
age_onset	15.4649	15.4790	-0.0087	1.1279	0.0336	0.1242	0.8318
index_sentence	40.0535	38.9775	0.0788	0.5688	0.0925	0.2450	1.1039
lsi_score	25.3063	26.0601	-0.1062	0.5819	0.0602	0.2230	0.9131

Percent Balance Improvement:

	Std. Mean Diff.	Var. Ratio	eCDF Mean	eCDF Max
distance	96.5	90.0	89.4	79.1
german_nationality	81.6	.	81.6	81.6
migrant_background	97.7	.	97.7	97.7
school	-124.0	.	-124.0	-124.0
criminal_record	44.0	.	44.0	44.0
index_homicide	-11.1	.	-11.1	-11.1
index_robbery	84.1	.	84.1	84.1
index_assault	99.1	.	99.1	99.1
index_sex	27.1	.	27.1	27.1
age_index	78.6	59.8	21.3	7.3
age_onset	97.4	64.3	37.8	14.1
index_sentence	49.5	-72.9	10.1	-6.5
lsi_score	60.5	22.0	14.0	-35.1

Sample Sizes:

	Control	Treated
All	38.	73.
Matched (ESS)	27.6	64.58
Matched	38.	73.
Unmatched	0.	0.
Discarded	0.	0.

Love plot

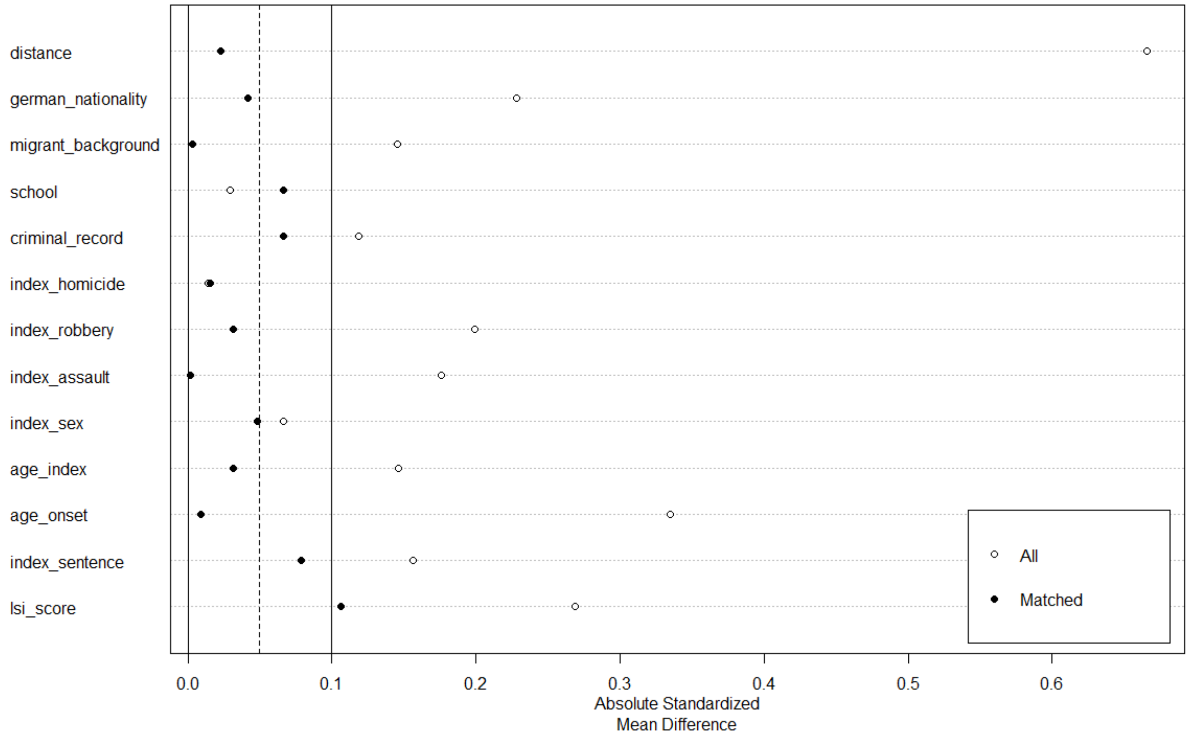
distance
german_nationality
migrant_background
school
criminal_record
index_homicide
index_robbery
index_assault
index_sex
age_index
age_onset
index_sentence
lsi_score
All
Matched
0.0
0.1
0.2
0.3
0.4
0.5
0.6
Absolute Standardized
Mean Difference

Empirical quantile-quantile (eQQ) Plots

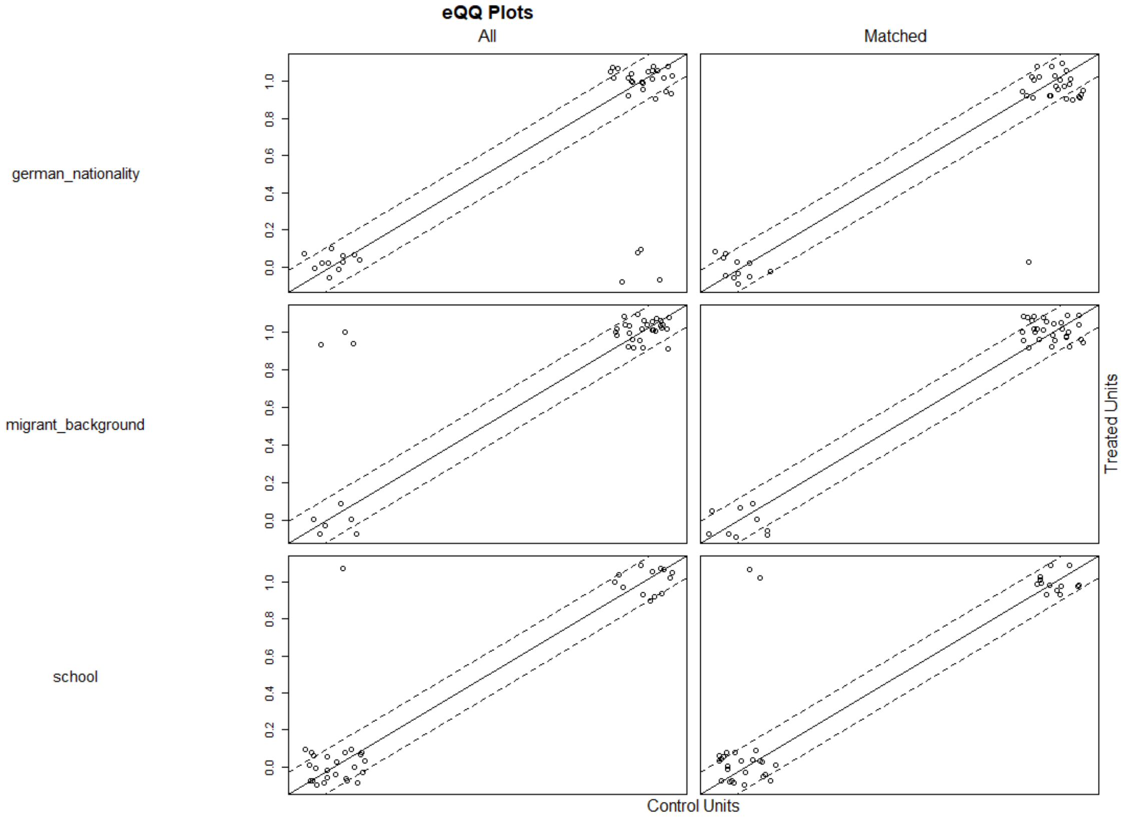

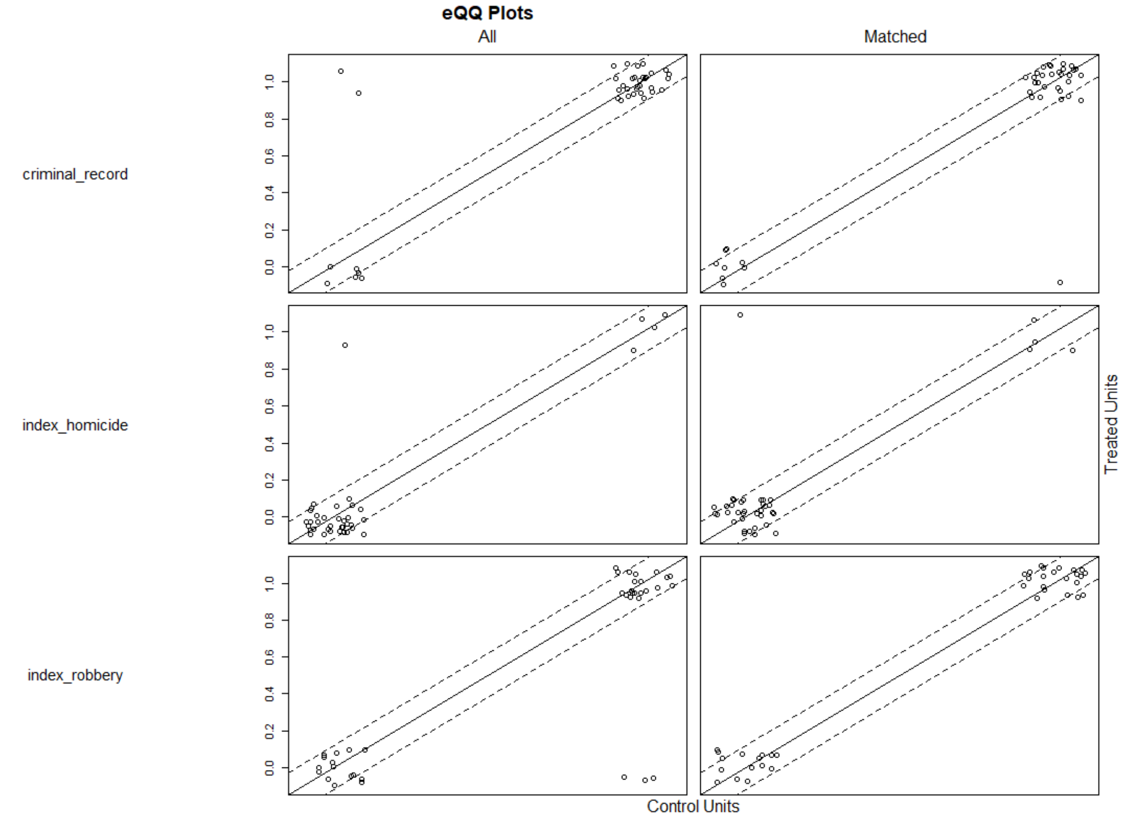

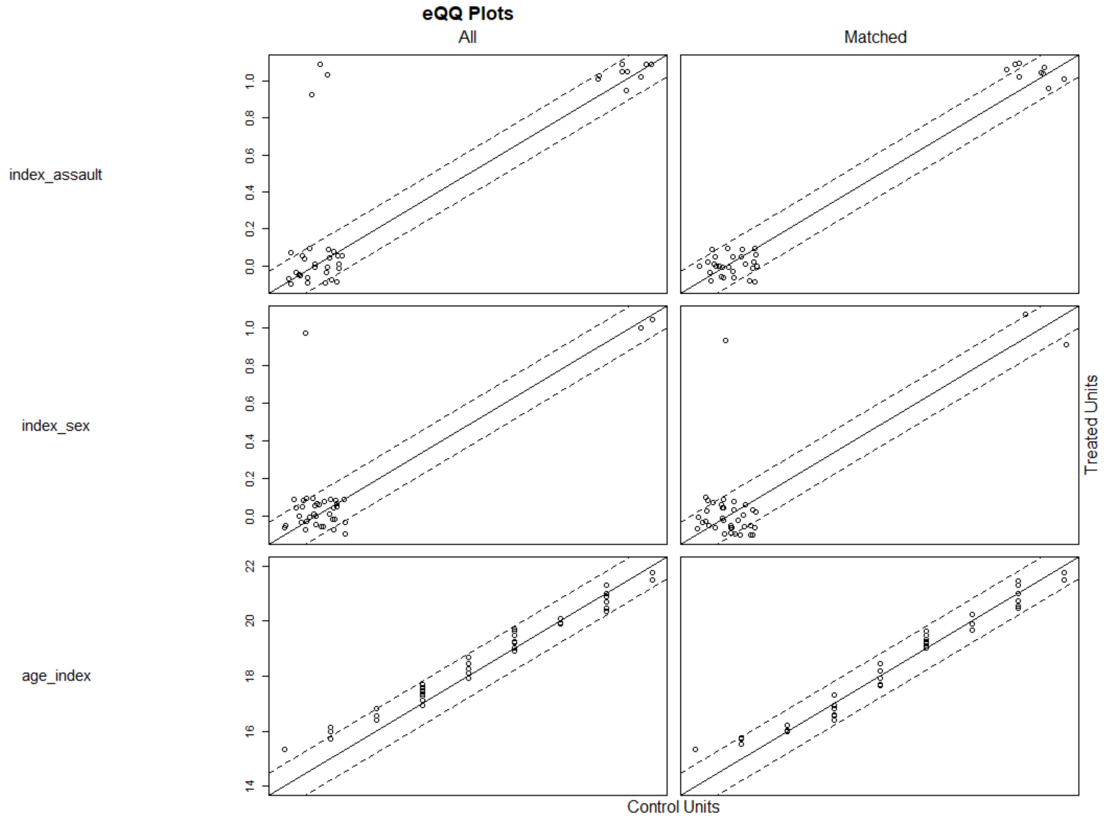
eQQ Plots
All
Matched
index_assault
index_sex
age_index
Treated Units
Control Units

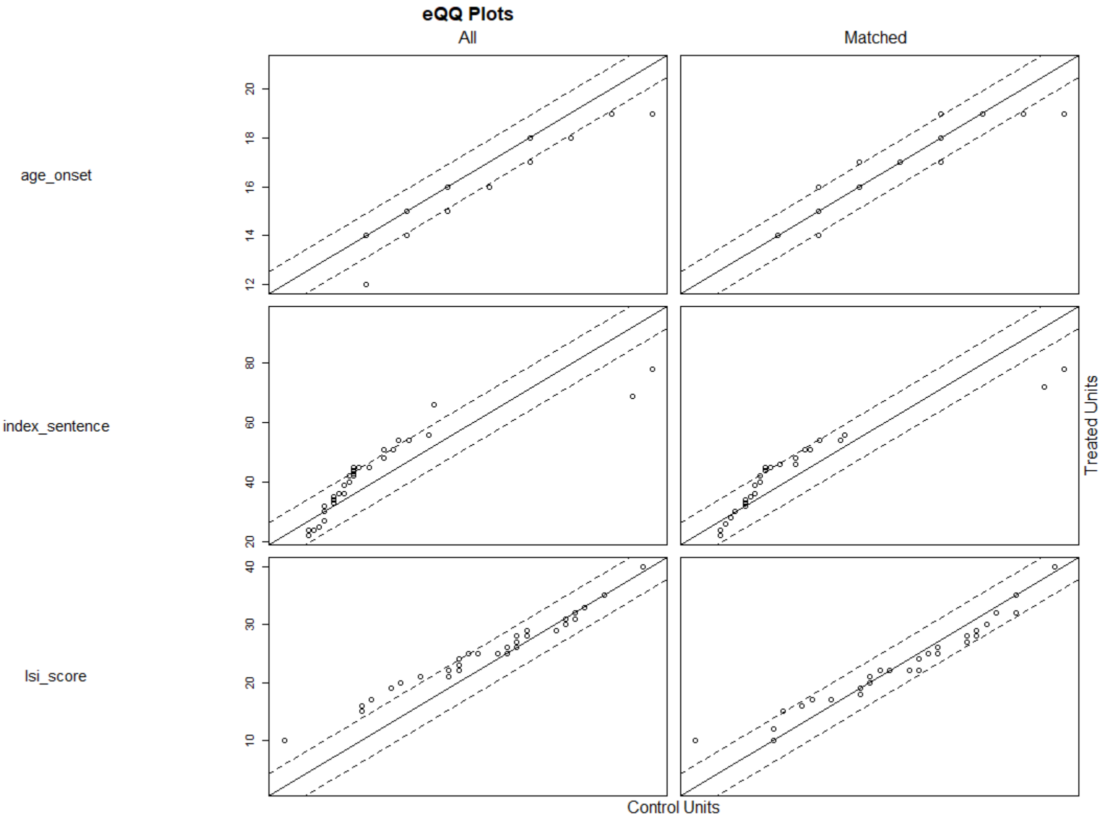
eQQ Plots
All
Matched
age_onset
index_sentence
lsi_score
Treated Units
Control Units

Empirical cumulative density functions (eCDFs)

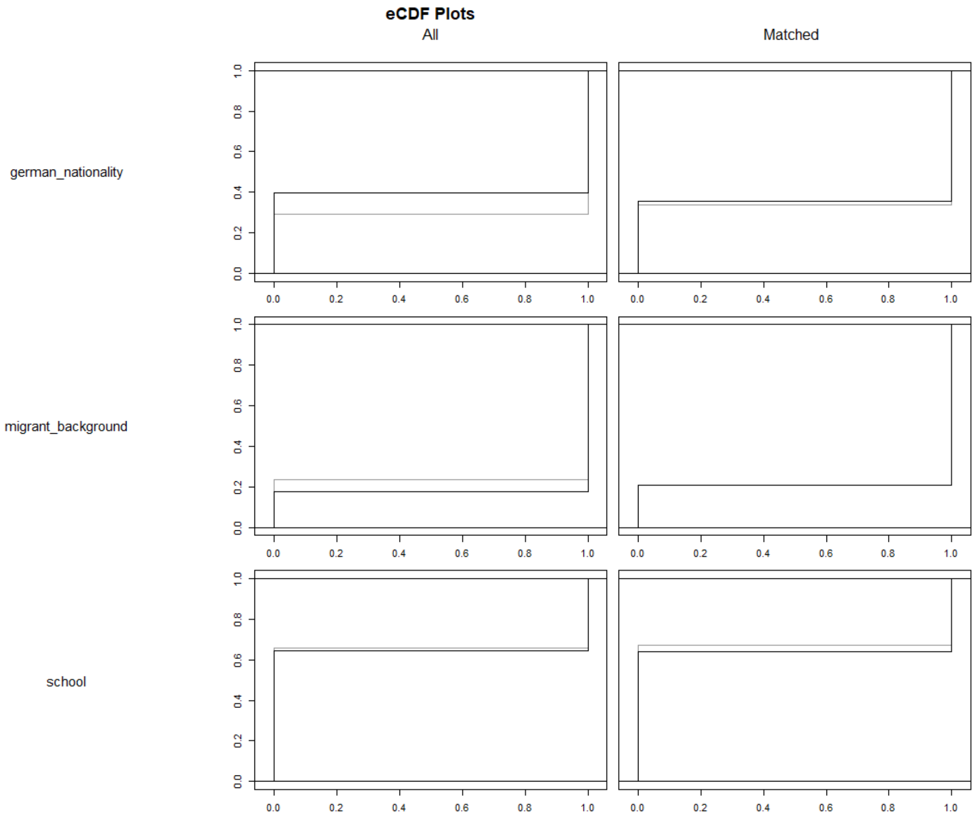

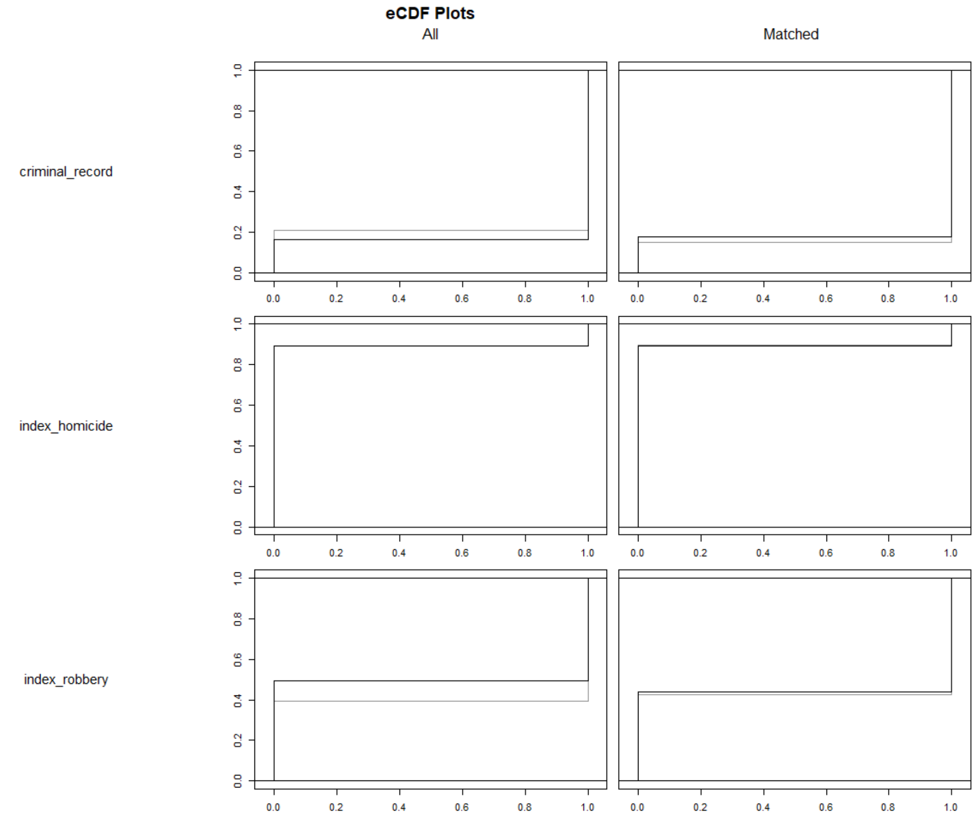

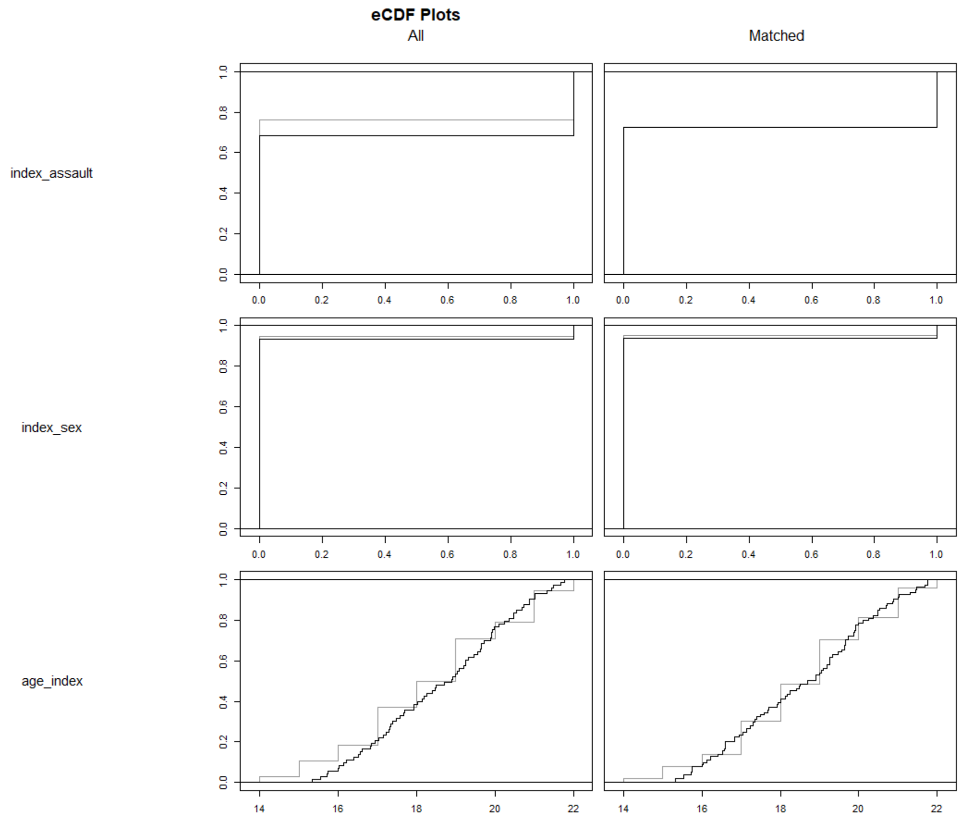
eCDF Plots
All
Matched
index_assault
index_sex
age_index

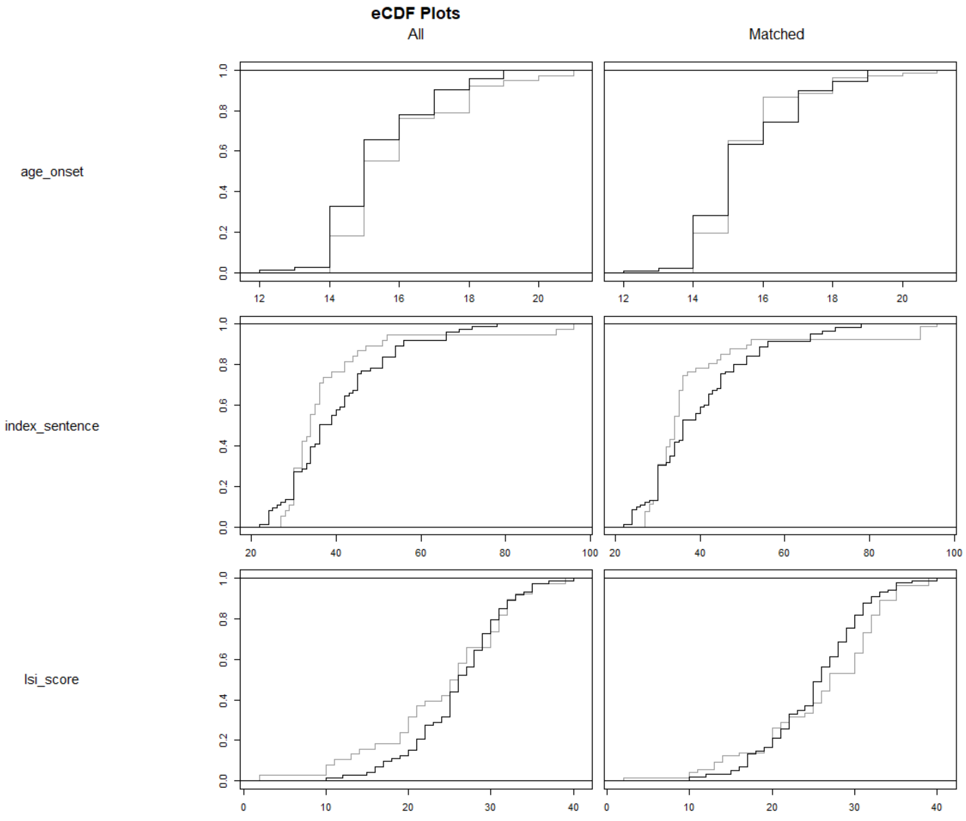
eCDF Plots
All
Matched
age_onset
index_sentence
lsi_score

Density plots

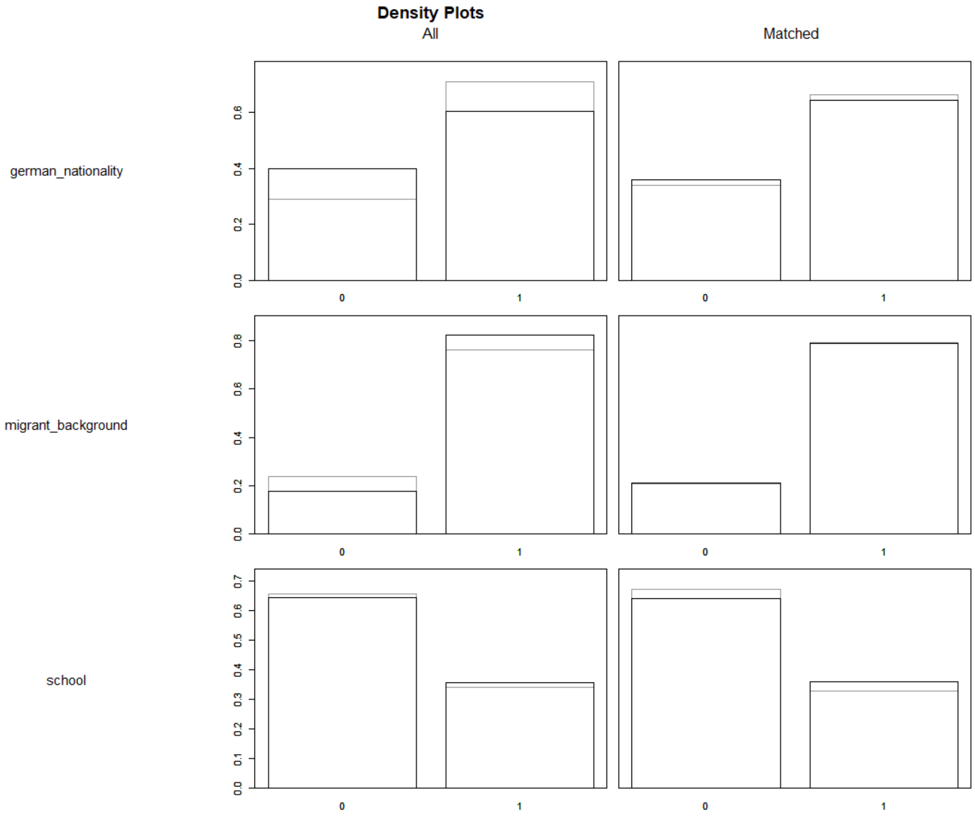

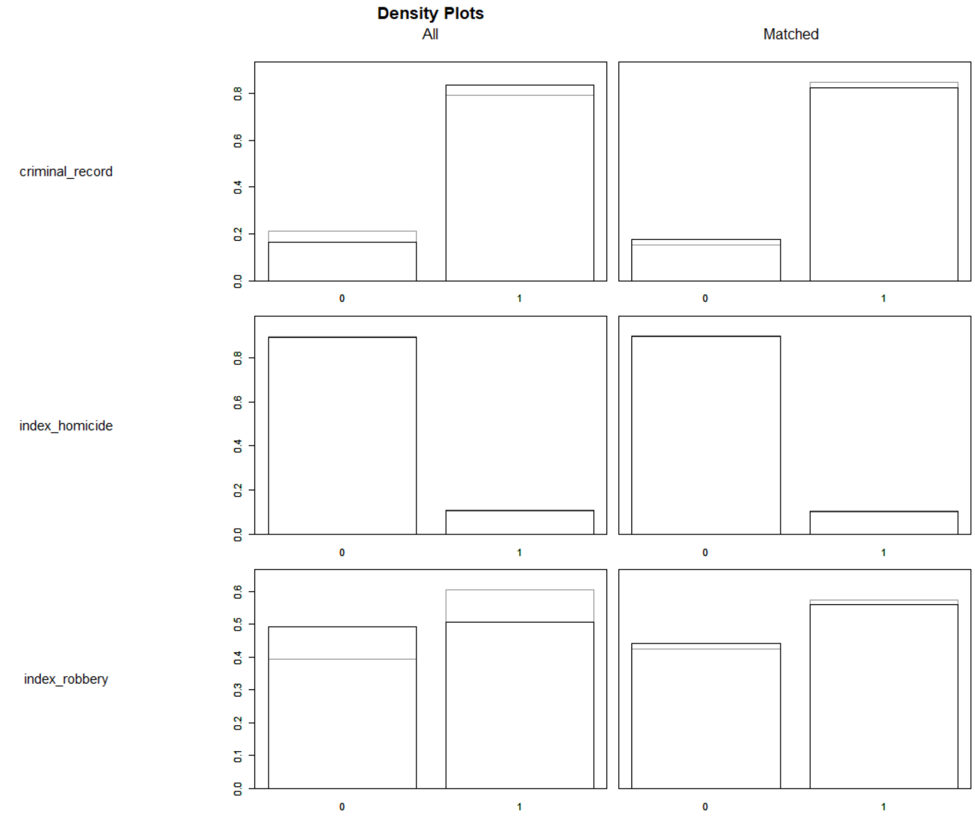

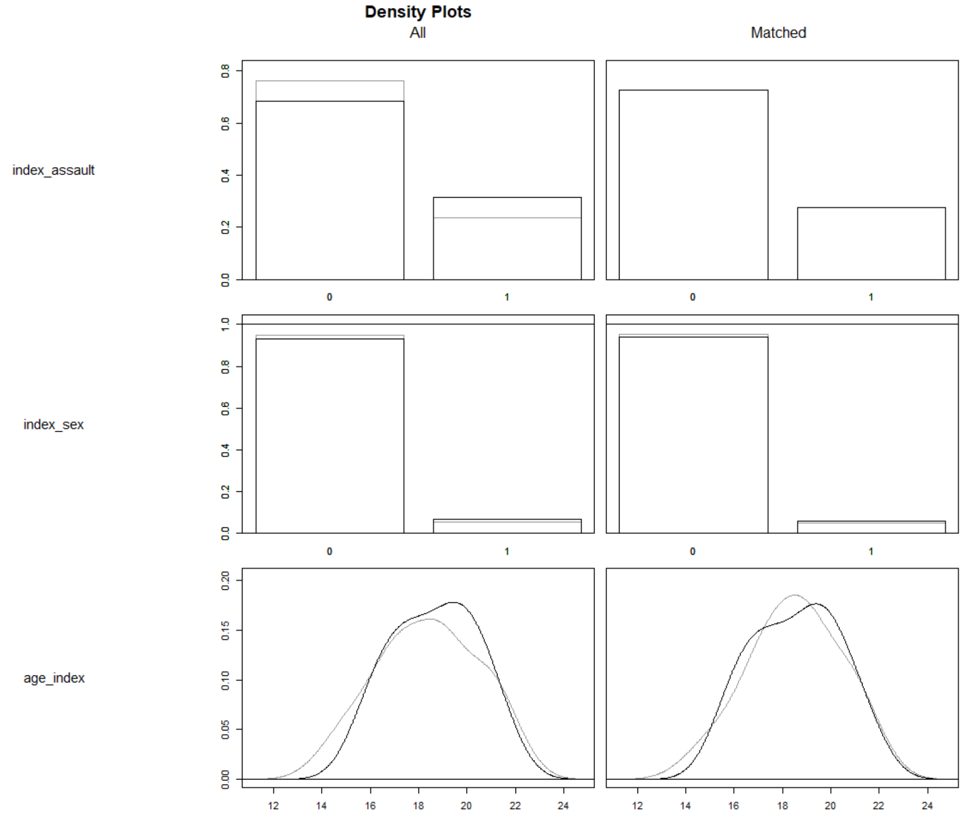
Density Plots
All
Matched
index_assault
index_sex
age_index

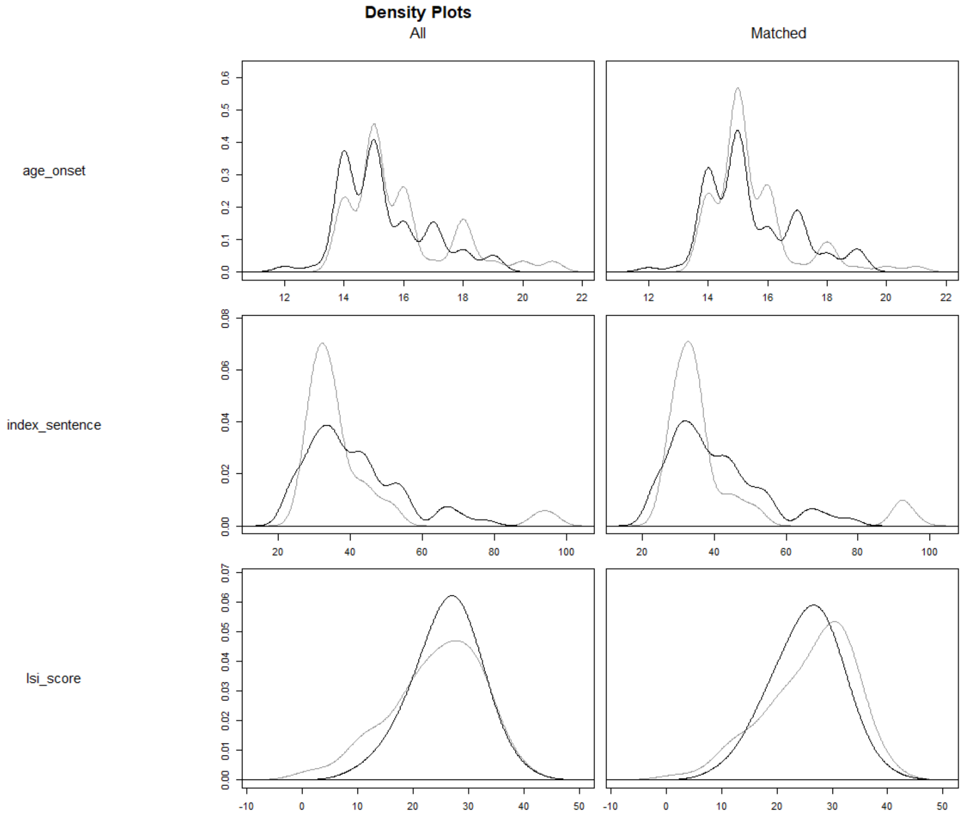
Density Plots
All
Matched
age_onset
index_sentence
lsi_score

ESM 2: Testing proportional hazards assumption in Cox regression models

Univariate Cox: Schönfeld residuals for non-violent/-sexual recidivism: $\chi^2(1) = 0.27$, $p = .6$

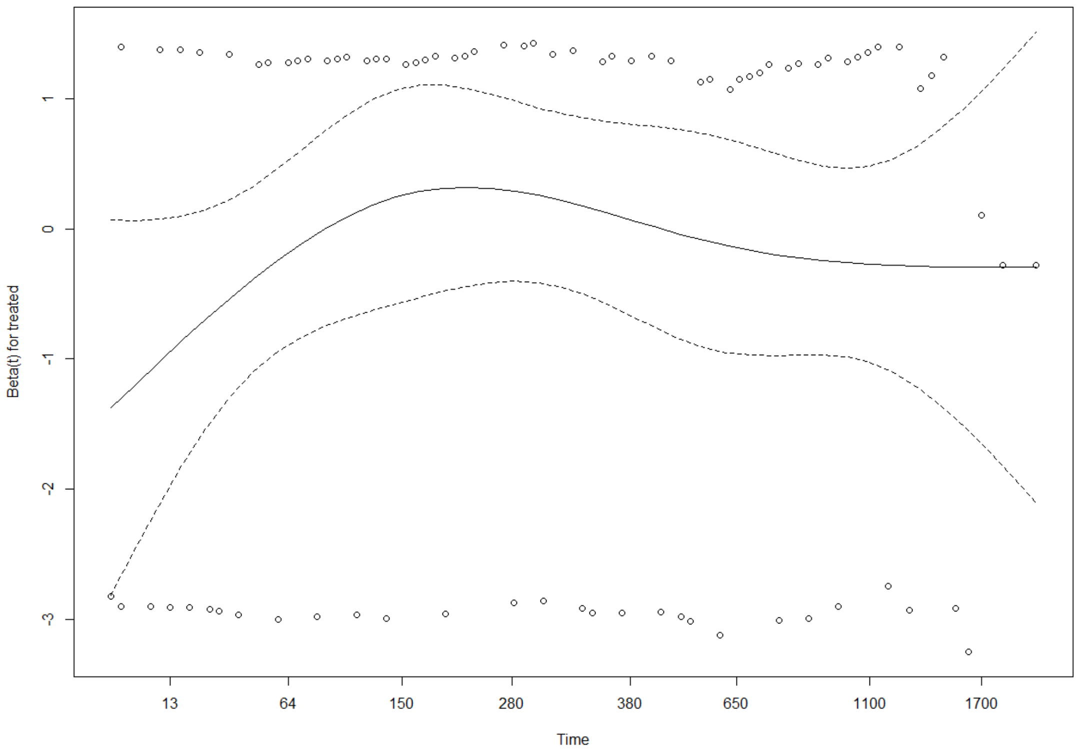

Univariate Cox: Schönfeld residuals for violent/sexual recidivism: $\chi^2(1) = 0.04$, $p = .84$

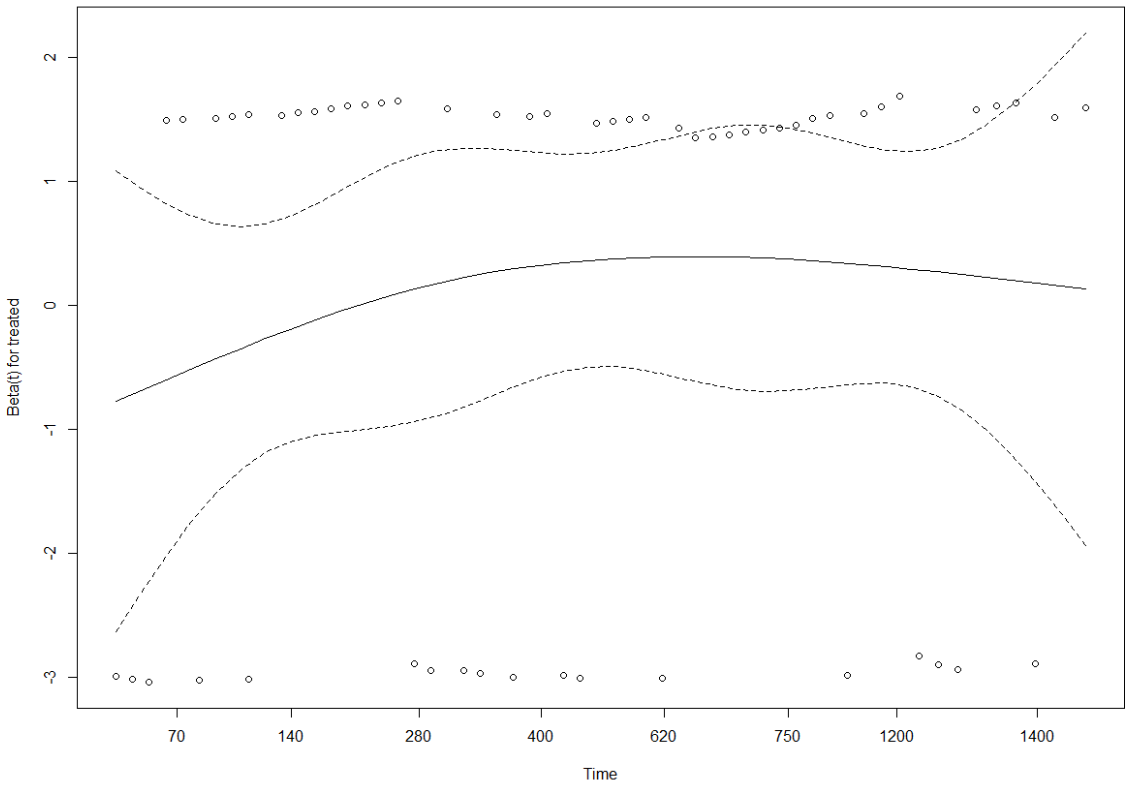

Multivariate cox: Schönfeld residuals for non-violent/-sexual recidivism: $\chi^2(7) = 9.56$, $p = .21$

	chisq	df	p
school_training	0.80079	1	0.37
vocational_training	5.37153	1	0.02
treatment_psychological	0.75817	1	0.38
treatment_rr	0.00678	1	0.93
treatment_social_skills	0.24741	1	0.62
treatment_violence_prevention	0.03163	1	0.86
treatment_addiction	0.69985	1	0.40
GLOBAL	9.56378	7	0.21

Multivariate: Schönfeld residuals for violent/sexual recidivism: $\chi^2(7) = 4.38$, $p = .74$

	chisq	df	p
school_training	0.0679	1	0.79
vocational_training	0.0945	1	0.76
treatment_psychological	2.2940	1	0.13
treatment_rr	2.6469	1	0.10
treatment_social_skills	0.2764	1	0.60
treatment_violence_prevention	0.1436	1	0.70
treatment_addiction	0.1281	1	0.72
GLOBAL	4.3767	7	0.74
